# A novel larynx‐preserving pharyngectomy technique using a thyroid gland flap after chemoradiotherapy

**DOI:** 10.1002/ccr3.2477

**Published:** 2019-10-02

**Authors:** Tetsuya Ogawa, Hiroki Okamoto, Daisuke Inukai, Rui Sano, Nobuyuki Katahira, Syunpei Yamanaka, Kinga Yo, Taichi Kan, Hiromi Ueda

**Affiliations:** ^1^ Department of Otorhinolaryngology Aichi Medical University Aichi Japan

**Keywords:** arytenoid reconstruction, chemoradiotherapy, head and neck surgery, larynx‐preserving pharyngectomy, thyroid gland flap

## Abstract

This case report describes larynx‐preserving pharyngectomy after chemoradiotherapy using a thyroid gland flap. A thyroid gland flap has good blood supply and reconstruction can be done in the same surgical field. The thyroid gland flap has potential as a novel appropriate flap for use in head and neck surgery.

## Graphical abstract

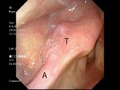

## 1 INTRODUCTION

Surgical resection for hypopharyngeal cancer is a difficult and challenging procedure. The hypopharynx has important functions in swallowing and normal voice function. These important functions can be adversely affected by structural damage caused by surgical intervention at this site, and therefore chemoradiotherapy for hypopharyngeal cancer has recently become widely accepted as an option for complete cure.^1^ Chemoradiotherapy has also been used to ensure functional preservation in esophageal cancer. Although chemoradiotherapy is considered definitive treatment for cancer, there is still some risk of relapse or recurrence of newly developed tumor in the same chemoradiotherapy field. For such cases, surgery is the only curative modality and is referred to as “salvage surgery”. Chemoradiotherapy is typically of very high intensity, and sometimes the field of irradiation might be damaged. Performing salvage surgery should be considered a difficult and dangerous procedure. Also, salvage surgery for hypopharyngeal cancer previously treated with chemoradiotherapy is very challenging. If the tumor is located on the arytenoid, the surgical procedure will be more complex and the risk even higher because complete resection of the arytenoid will be needed with subsequent reconstruction to prevent aspiration.

Here, we encountered a case of hypopharyngeal cancer arising from the right arytenoid where the patient had been previously treated with chemoradiotherapy for esophageal cancer. We completely resected the affected arytenoid and performed reconstruction with a thyroid gland flap, which typically has sufficient blood supply. We report a novel salvage larynx‐preserving pharyngectomy technique using a thyroid gland flap for arytenoid reconstruction.

## 2 METHODS

### 2.1 Clinical findings and treatment strategy

This 58‐year‐old man with a history of sore throat had received chemoradiotherapy for esophageal cancer 6 years earlier at an irradiation dose of 61.6 Gy with cisplatin (100 mg/kg and 5FU 1000 mg/kg twice. The irradiation field included the hypopharyngeal area.

**Figure 1:**
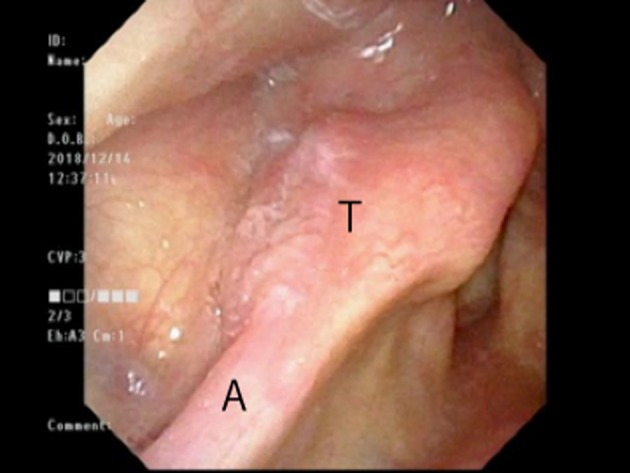
Tumor spread extending to the right arytenoid pyriform sinus. A, arytenoid; T, tumor

Endonasal flexible fiberoptic endoscopy revealed a white irregular area that was mainly on the right arytenoid with extensive spread to the pyriform sinus (Figure 1). Biopsy revealed the tumor as squamous cell carcinoma. PET‐CT and enhanced cervical CT revealed no cervical lymph node metastasis or distant metastasis. The final diagnosis was hypopharyngeal cancer T1N0M0. Notably, the patient had received high‐intensity chemoradiotherapy 6 years earlier because the cancer was diagnosed early. In light of this, our radiation oncologist determined that radiotherapy would not be curative. Also, our endoscopists thought that the tumor might have invaded the deep parts of the arytenoid mucosa and predicted that due to the positioning, it would be difficult to resect the whole tumor with adequate surgical margins. Accordingly, they decided that endoscopic resection was not appropriate.

We therefore decided to use an external approach with salvage larynx‐preserving pharyngectomy and arytenoid reconstruction. This surgery is advantageous in that it allows for direct removal of the tumor with an adequate surgical margin. However, this external approach also poses a risk of complications like chondrites and necrosis, which would cause severe total necrosis of the hypopharynx and larynx and increase the possibility of total pharyngolaryngectomy. The most important considerations are complete resection with arytenoid reconstruction, good blood supply, and permanent adequate arytenoid height. To achieve this, we developed a novel approach using a thyroid gland flap that typically has good blood supply from the superior thyroid artery. The thyroid gland flap is a local flap, and this is beneficial for this patient as a minimally invasive surgery. The surgical field is restricted, and the surgical intervention is minimum, and this is its value as salvage surgery. This flap can be used to confirm the arytenoid height using thyroid cartilage, and then the site is covered with the blood supply flap.

### 2.2 Surgical Plan

**Figure 2:**
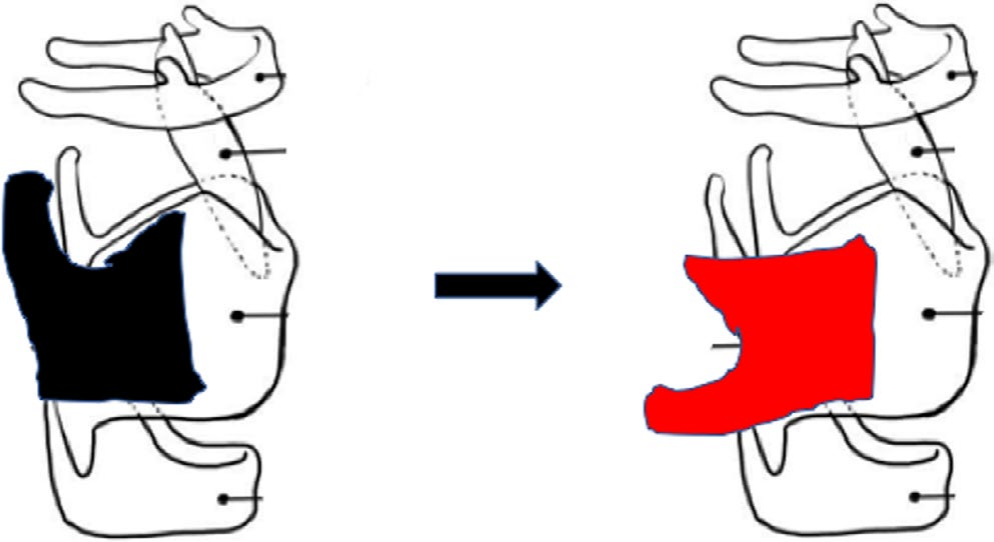
Left figure shows the cutting of the thyroid cartilage. Right figure shows the removed thyroid cartilage is refitted on to the cricoid cartilage at 90‐degrees counterclockwise to get the exact height

We initially planned the salvage larynx‐preserving pharyngectomy as follows:

1. Skin flap elevation via a left convex incision. This left convex top skin flap is a full‐thickness flap because it will be inserted to the larynx to cover the hypopharyngeal mucosal defect.
2. Preservation of the internal branch of the superior laryngeal nerve.
3. Cutting and reuse of thyroid cartilage. Scheme of cutting the cartilage and technique for reuse of the cut cartilage and replacement on the cricoid cartilage (Figure 2).
4. Wide tumor resection area with adequate surgical margin, and frozen sections obtained for histopathological analysis.
5. Reconstruction of the mucosal defect using convex skin. Total coverage of the mucosal defect with skin followed by cartilage placement and fixation by the building up to the height of the arytenoid.
6. Thyroid gland flap with appropriate size adjustment, and then placement over the surgical defect to cover up the area.
7. Laryngeal suspension.

### 2.3 Surgical procedure

**Figure 3:**
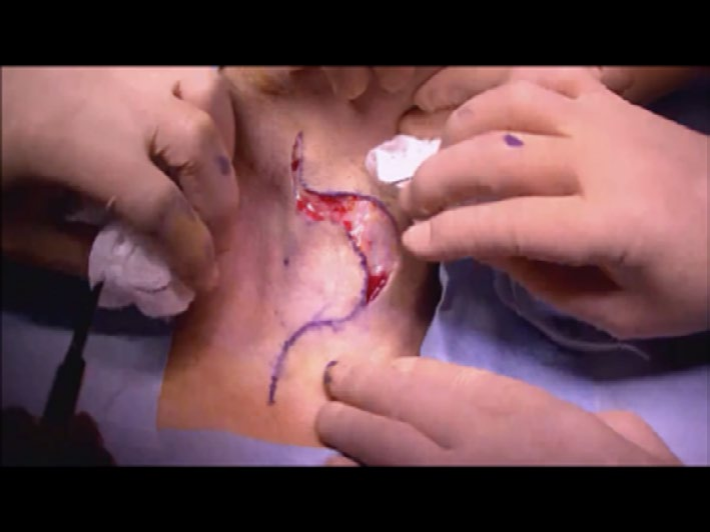
Convex skin incision lines on the left. The skin flap was elevated with superficial fascia and cervical muscles as full thickness

**Figure 4:**
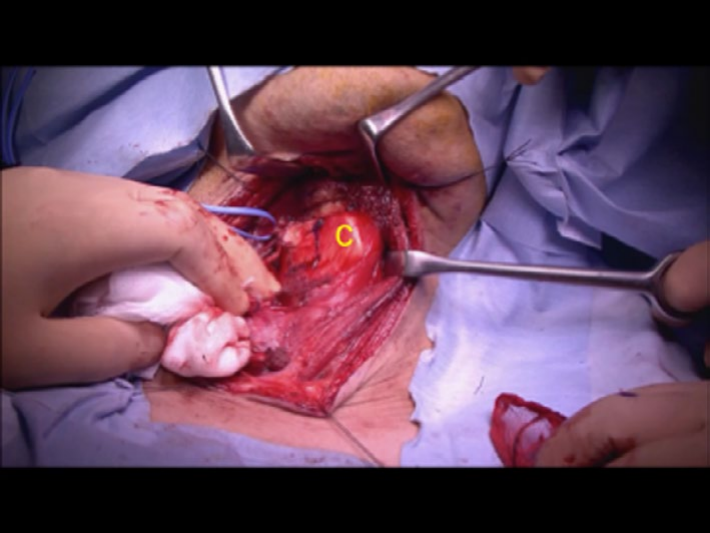
The thyroid cartilage incision placed to cover two‐fifths of the right side of thyroid cartilage and one‐fifth of the caudal side. C, thyroid cartilage

**Figure 5:**
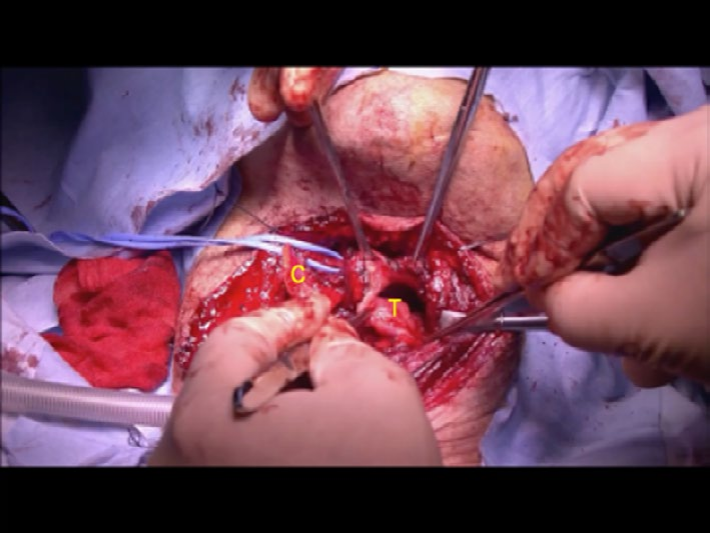
Pharyngeal tumor is resected with the surgical margin including the arytenoid cartilage. C, thyroid cartilage. T, tumor

The patient was placed in the supine position. Airway intubation was initially performed via the oral cavity. The skin incision was placed as shown in Figure 3. After elevation of the skin flap, the bilateral sternohyoid muscles were preserved. The right muscle could be used to cover the surface of the reconstructed area. The right sternothyroid muscle was displaced so the thyroid cartilage could be viewed directly. Next, the internal branch of the superior laryngeal nerve was identified and preserved to maintain intact internal laryngeal sensation, which would allow for adequate swallowing function. The thyroid cartilage was cut using a scalpel as shown in Figure 4. This incision line covered two‐fifths of the right side of the thyroid cartilage vertically and one‐fifth of the caudal side horizontally. At this point, the inner aspect of the larynx could be seen. The paratracheal tissue was then cut to allow access to the hypopharyngeal area. Then the main tumor on the right arytenoid is shown in Figure 5. On achieving a 1‐cm surgical margin around the main tumor, the pharyngeal tumor was removed with the arytenoid cartilage en bloc to ensure adequate surgical margins. Frozen sections were prepared and histopathological analysis confirmed that the tumor resection was complete.

For the reconstruction, a local flap and tissue were deemed best for the patient in terms of minimally invasive surgery because he had previously received high‐intensity chemoradiotherapy for esophageal cancer at the same site. The tissue around the area was fragile and had poor blood supply. We thus decided to use a thyroid gland flap from the right lobe. This salvage larynx‐preserving pharyngectomy required thorough reconstruction of the arytenoid to ensure preserved swallowing function postoperatively. Thus, the permanent height reconstruction had to be recreated as near normal as possible. We therefore used donor thyroid cartilage and refitted this to the cricoid cartilage at 90‐degrees counterclockwise to get the exact height (Figure 2). The cricoid cartilage was fixed by suturing with 2‐0 absorbable suture to the contralateral residual thyroid cartilage. However, there was some dead space at the operative site, because of the surgical intervention and removal of tissue. This dead space could lead to local infection with severe complications and needed to be filled by placing a flap that would ensure adequate blood flow for the reconstructed thyroid cartilage and skin. So, we decided to use a right thyroid gland flap with the superior thyroid artery intact.

**Figure 6:**
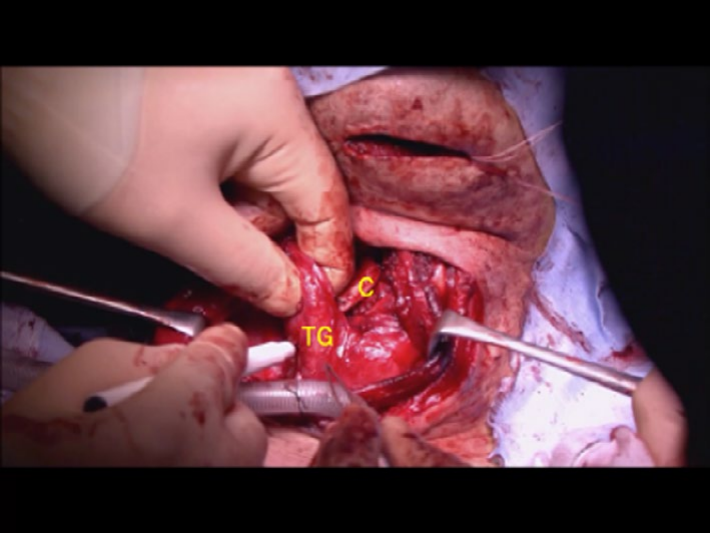
The upper area of the thyroid gland is shown.C, thyroid cartilage (refitted); TG, thyroid gland

**Figure 7:**
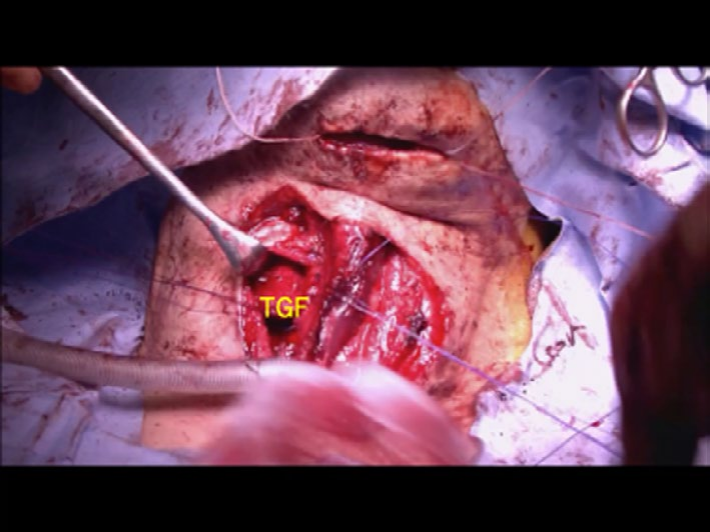
The thyroid gland flap was placed on the defect over the reconstructed cartilage. TGF, thyroid gland flap

The upper area of the thyroid gland flap is shown in Figure 6, and it traced proximally. There was no need to trace the artery completely because it was readily mobile and ran on the surface of the surgical defect. The thyroid gland flap was cut to adjust the size to fit the defect area. The flap was then placed on the defect over the reconstructed cartilage (Figure 7). The internal branch of the superior laryngeal nerve was completely preserved. Laryngeal suspension was also performed to facilitate recovery of swallowing function. The surface of the surgical site was then covered with the sternohyoid muscle, and the skin flap was closed. Total blood loss was 177 mL, and the total operative time was 4 hours 17 minutes.

## 3 RESULTS

### 3.1 Pathology report

The pathology report revealed that all the surgical margins were negative but the tumor had invaded into the deep mucosa. Based on our findings in this report, the outcome of this procedure was adequate for this patient.

### 3.2 Postoperative course

The postoperative course was uneventful and oral intake resumed on postoperative day (POD) 15. There was some infection of the right side of the neck, which was managed successfully with conservative therapy. Decannulation was performed by POD 47, and the patient resumed a full normal diet by POD 65 and was discharged from hospital. Voice was normal, and maximum phonation time was 19, 21, and 18 seconds.

After the procedure, the patient resumed normal diet within a short time, which was the same as before the operation. There was no sign of recurrence as of 7 months postoperatively.

### 3.3 Video fluoroscopic swallowing exam

**Figure 8:**
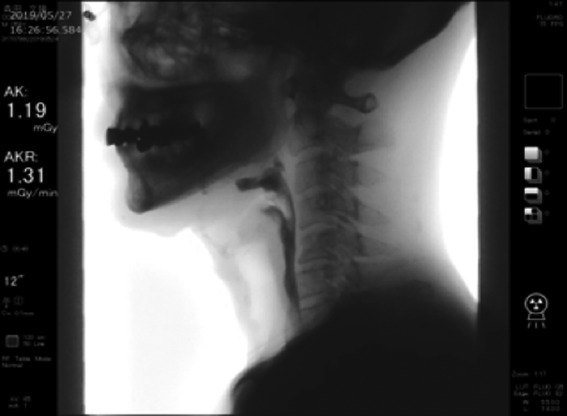
VFSE showing no impairment of swallowing beyond the arytenoid fold

Swallowing was found to be normal and adequate; video fluoroscopy is shown in Figure 8. The bolus moved mainly on the left side but also on the right side, which was the reconstructed side. VFSE showed there was no swallowing dysfunction beyond the arytenoid fold.

### 3.4 Endoscopic findings

**Figure 9:**
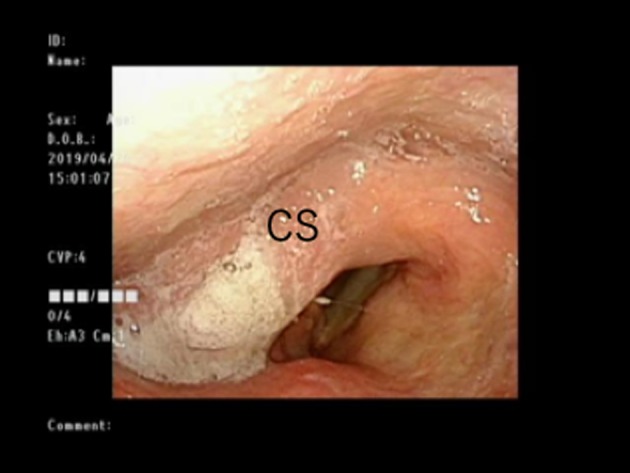
Reconstructed mucosa using the skin completely is adjusted to cover the pharynx and larynx. CS, Convex skin flap

Nasal endoscopic findings of the larynx and pharynx are shown in Figure 9. The reconstructed mucosa covered by the skin flap was well positioned on the pharynx and larynx. The height of the arytenoid was appropriate, which would serve to prevent aspiration. Also, this height was permanent because of the reconstruction with the cartilage and the thyroid gland flap with an abundant blood supply.

### 3.5 Thyroid gland flap location and function

**Figure 10:**
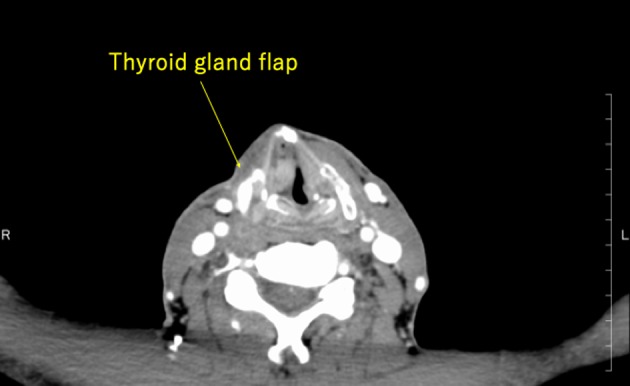
Enhanced CT showing that the thyroid gland flap is accurately placed to cover the defect, and the surgical area is clearly enhanced

Regarding thyroid gland flap status enhanced CT revealed that the flap was adequately positioned on the defect at the surgical area, and this was clearly enhanced on CT (Figure 10). Thyroid function was normal. Taking together the absence of severe postoperative complications and CT findings, we judged that the adaptation of this thyroid gland flap with blood supply by the superior thyroid artery was optimal and was suited to the defect area after chemoradiotherapy.

## 4 DISCUSSION

The head and neck region are structurally complex and contains sites that serve vital functions. The treatment choice for head and neck cancer is difficult because of the trade‐off between complete cancer cure and functional preservation.^2^ For these reasons, chemoradiotherapy has recently been widely used to achieve complete cure of head and neck cancer^1^ as well as for esophageal cancer,^3^ because the need for surgical intervention is limited.^1^ The surgical approach is precise but sometimes leads to severe adverse effects.^4^ So, these cancers now are being treated not only with surgery but also with chemoradiotherapy. Chemoradiotherapy is a radical treatment modality, but in some cases relapse does occur or sometimes a newly developed tumor arises on the previously irradiated field.

If the cancer occurs or recurs in the same radiation field, salvage surgery is the only option because further treatment with radical irradiation is no longer possible. The surgery will require adequate surgical margins because of the radical salvage procedure. Sometimes, the procedure requires a wider surgical field with direct view of the tumor area to ensure adequate margins. However, because the surgical site has been damaged by previous high‐intensity chemoradiotherapy, further surgical intervention will lead to severe complications such as chondrites and necrosis.^5^ There is also a risk of low blood supply because of vascular damage by chemoradiotherapy.^6^

In terms of the head and neck region, resection of tumors arising from the hypopharynx tends to be difficult because the hypopharyngeal region has a complex anatomy and is closely related to voice and swallowing. Thus, salvage larynx‐preserving pharyngectomy will be challenging for the head and neck surgical oncologist.^7^, ^8^, ^9^ If the tumor is discovered at an advanced stage, radical total pharyngolaryngectomy and free flap reconstruction could be an acceptable option for both the patient and surgeon. Also, if the tumor occurs on the posterior aspect or at a site where resection is easy, this can be done under endoscopic or video‐guidance so as to preserve function.^10^ However, if the tumor is on one side of the arytenoids, this will be difficult. Because the arytenoids are firm and compact structures, an adequate height for surgery is needed with a sufficient blood supply flap. Thus, salvage larynx‐preserving pharyngectomy could be difficult. Until now free flap reconstruction was done as salvage larynx‐preserving pharyngectomy. In terms of reconstruction of the arytenoid height, some reports have described using local tissue for free flap reconstruction. Yamawaki et al reported that hyoid bone free transfer and radial free forearm reconstruction was successful in laryngopharyngectomy.^11^ However, this method requires a free forearm flap to cover the hypopharyngeal defect, so it will take longer to complete and incur more cost. Also, it requires a wider surgical field, which then poses some risk to the remaining thyroid cartilage and cricoid cartilage, especially in this case as the patient had received high‐intensity chemoradiotherapy.

We think that salvage surgery should be done within a minimally invasive surgical field and the surgical field should be manipulated as little as possible, and only near the neck. The reconstruction procedure should be done using tissue with a good blood supply to prevent surgical infection via contaminated thyroid cartilage and/or a transferred skin flap. These requirements highlight the need for a new optimal flap technique, and therefore we opted for a novel approach to salvage larynx‐preserving pharyngectomy. We therefore chose to use a thyroid gland flap to reconstruct the surgical defect because adequate blood supply was likely after larynx‐preserving pharyngectomy. We have previously used the thyroid gland flap for salvage vertical partial laryngectomy.^12^ The thyroid gland flap was harvested from the superior aspect of the designated thyroid lobe, which is directly supplied by the superior thyroid artery. The thyroid gland flap is cut and the size adjusted to fit the surgical defect. There is no need to totally displace and use one side of the thyroid gland. Also, there is no need to view the recurrent laryngeal nerve because the superior aspect is easy to free from the cricoid cartilage above. Next, the cut and adjusted thyroid gland flap is rotated smoothly and moved onto the surgical defect site. There is no need to move the superior thyroid artery proximally. There is only the need to dissect and remove the artery until the thyroid gland flap just fits onto the surgical defect, and adequate blood supply would be expected via the superior thyroid artery, which is the first branch of the external carotid artery. This site of thyroid gland flap elevation is very close to the site of head and neck surgery, so the extent of surgical intervention is mild. Also, operative time and cost are minimal compared with conventional pharyngolaryngectomy because raising the thyroid gland flap is the same procedure done in the same field. Compared with the free flap, the operative time and extent of intervention is low. This then lowers the possibility of complications.

Although this case was a difficult salvage surgery, there were no severe complications aside from an infection of the skin of the neck, which did not cause severe damage and the clinical course was otherwise good. CT revealed the true reason for this. Enhanced CT showed that the thyroid gland flap was accurately located on the free reconstructed thyroid cartilage. Compared with nonenhanced CT, enhanced CT showed that the thyroid cartilage was fixed between the flap and the cricoid cartilage, as well as the reconstructed skin. Thus, the flap was suitable and blood flow was adequate, so if there was any local infection this blood supply would ensure high local drug concentration, causing no further complications and preventing dehiscence. This indicates that adjusting the size of the thyroid gland flap is necessary for salvage larynx‐preserving pharyngectomy.

This technique resulted in an arytenoid with an appropriate height due to adequate reconstruction on a free resized thyroid cartilage supported by a sufficient blood supply flap, which is the thyroid gland flap. This facilitated good swallowing function and prevented aspiration. The patient resumed a normal family diet within the normal expected time. Moreover, the patient's voice has not changed after the surgery; voice assessment was rated G0 R0 B0 A0 S0 on the Grade, Roughness, Breathiness, Asthenia, Strain (GRBAS) scale of the Japan Society of Logopedics and Phoniatrics, and the maximum phonation time was 18, 20, and 19 seconds. This is because the larynx‐preserving pharyngectomy procedure did not involve dissection of the vocal cords even though the reconstruction of the arytenoid part will extend the cord directly. Thus, in this case, voice function is well preserved.

Endoscopic findings showed that the transferred skin was well adapted to the arytenoid mucosa and that the skin covered the entire reconstructed area and connected with the laryngeal mucosa. The shape and height of the right arytenoid were the same as that of the left. Video fluoroscopy showed that the bolus ran mostly in the left side. Clearance and speed were excellent and there was no aspiration. This is because of the successful arytenoid reconstruction and its adequate height.

We accurately preserved the internal branch of the superior laryngeal nerve and performed laryngeal suspension and fixation, procedures which might have influenced the good functional outcome. Finally, thyroid function was not impaired because in raising the thyroid gland flap, the thyroid lobe was not removed. Thus, using the thyroid gland flap was beneficial for surgery in this case.

In conclusion, chemoradiotherapy is being widely used for treatment of head and neck cancer as well as esophageal cancer. There will likely be cases of newly developed cancer or recurrence at the irradiation field. For such cases, the only definitive curative treatment is salvage surgery, which can only be performed by a surgical oncologist. While performing salvage surgery in the head and neck is critical and challenging, it is a necessary procedure. The thyroid gland flap is appropriate because of the sufficient blood supply, ease of adaptation to the head and neck defect, same surgical field, and functional preservation. Therefore, the use of thyroid gland flap for salvage surgery is expected to increase.

## CONFLICT OF INTEREST

This research did not receive any specific funding and all authors have no conflict of interest to declare.

## AUTHOR CONTRIBUTIONS

Tetsuya Ogawa, M.D: involved in operation concept and main operator. Hiroki Okamoto, MD, Daisuke Inukai, MD, Syunpei Yamanaka, MD Rui Sano, M.D: involved in operation assistance. Nobuyuki Katahira, MD, Kinga Yo, MD Taichi Kan, MD Organizer; Hiromi Ueda, M.D: involved in VFSE and voice checking.
